# Efficient Characterization of Tetraploid Watermelon

**DOI:** 10.3390/plants8100419

**Published:** 2019-10-16

**Authors:** Na Zhang, Yaning Bao, Zhouli Xie, Xing Huang, Yuhong Sun, Gang Feng, Hongxia Zeng, Jian Ren, Yuhua Li, Jianshun Xiong, Wei Chen, Chao Yan, Mi Tang

**Affiliations:** 1 Institute of Crop Science, Wuhan Academy of Agricultural Sciences, Wuhan 430345, China; zhangna@wuhanagri.com (N.Z.); sunyh68@163.com (Y.S.); zenghongxia@wuhanagri.com (H.Z.); renjian@wuhanagri.com (J.R.); lyh401129@126.com (Y.L.); duan8819@sina.com (J.X.); whsnks123@126.com (W.C.); 2 College of Plant Science and Technology, Huazhong Agricultural University, Wuhan 430070, China; baoyaning@webmail.hzau.edu.cn; 3 Department of Genetics, Development and Cell Biology, Iowa State University, Ames, IA 50011, USA; xzhouli@pku.edu.cn; 4 Environment and Plant Protection Institute, Chinese Academy of Tropical Agricultural Sciences, Haikou 571101, China; feng8513@sina.com (G.F.); chaoyan@catas.cn (C.Y.)

**Keywords:** watermelon, diploid, tetraploid, flow cytometry, qPCR

## Abstract

Watermelon (*Citrullus lanatus* (Thunb.) Matsum. &Nakai) is an economic crop, which is widely cultivated around the world. The ploidy study of watermelon has an important role in field breeding and production, therefore, timely and convenient ploidy detection is necessary to accelerate its application. Traditionally, the ploidy of watermelon was determined by a series of time-consuming, expensive, and less efficient methods. In this study, we developed a more efficient method to simplify and accelerate the polyploidy identification in watermelons. We first confirmed the ploidy of watermelon by traditional tetraploid morphological features and well-established flow cytometry (FCM). Then we developed a reliable real-time quantitative PCR (qPCR) technique by quantifying the highly conserved 5S rDNA sequence and its copy numbers. This technique requires less sample collection and has comparable accuracy to FCM, it accelerates the analysis process and provides a new method for the identification of polyploidy of watermelon.

## 1. Introduction

Watermelon is an annual vine herb of the Cucurbitaceae watermelon species. It is one of the important economic crops which is widely cultivated for its sweet dessert fruit or “the king fruit in summer”. Typically, the diploid watermelon (2n = 2× = 22) is more common in nature, while the autopolyploids make the triploid watermelon (3n = 3× = 33) and tetraploid watermelon (4n = 4× = 44) possible [1]. The triploid watermelon is a hybrid generation of common diploid and tetraploid watermelon, and the triploid watermelon is famous for the high quality of seedless fruit. Autopolyploids have excellent characteristics such as diverse DNA contents, high secondary metabolite organisms, large tissues and organs, improved yield, and high tolerance to abiotic and biotic stresses [2,3]. For example, the tetraploid watermelon contains an enlarged leaf, thicker vine and peel, larger pistil and stamen flower organs, and bigger seeds. Additionally, the tetraploid watermelon has: a higher chlorophyll content; the total β-carotene, lycopene, fructose, and glucose content is higher than diploid [4,5]; and it is more tolerant to salt stress compared to diploid [6]. Although with respect to those benefits, the low frequency of autopolyploids and the obscure observation and selection limit the application to commercial cultivation.

Nowadays, the successful usage of colchicine-induced Datura tetraploid by Blakeslee and Avery in 1937 [1], promotes the artificial induction of polyploids. For instance, the tetraploid parents were produced with the treatment of colchicine on newly emerged diploid seedlings [7]. The tetraploid inbred line can also be propagated by seed and tissue culture [8,9,10]. After induction of polyploids, the observation and selection become more important. Several approaches have been used to identify ploidy level in watermelon. The most intuitive method is morphological characterization. Previous studies have found that leaf length and width, ovary diameter, male flower petals, and pollen sac diameter are good indicators for identification [11,12]. The number of chloroplasts in watermelon leaf guard cells is positively correlated with plant ploidy, which can be used as a rapid and effective method as well [13,14]. Chromosome counting is also a common method for determining ploidy, but the small size of the watermelon chromosome makes it difficult to achieve accurate results, and this method is not practical for non-dividing cells in differentiated tissues, such as leaves [15]. Therefore, an efficient and reliable ploidy measurement technique is required for accelerating the tetraploid breeding of watermelon. Commonly, chromosome number increases or decreases will result in the increase or decrease of nuclear DNA content, respectively. Flow cytometry (FCM) is a fast and efficient method for identifying ploidy by rapidly measuring the size of the nucleus and the total amount of DNA in the nucleus, which has been well established in watermelon [16,17,18,19,20]. It can apply to tissues grown in the field or in the greenhouse. But the choice of the organization is limited, tissues that are young and have poor cell division are better detected by FCM. In addition, conventional flow cytometers are expensive and not common in routine laboratories. qPCR is a mature method for gene expression detection and copy number analysis [21,22]. After ploidy induction, the chromosomes were doubled in single cells, which would inevitably double the copy number of genes [1]. Thus, we developed a qPCR method for watermelon ploidy detection that quantifies the copy numbers of the highly conserved 5S rDNA sequence, which was also compared with the FCM method to evaluate the efficiency of the new method.

## 2. Results

### 2.1. Tetraploid Induction and Morphological Characterization in Watermelon

**Figure 1:**
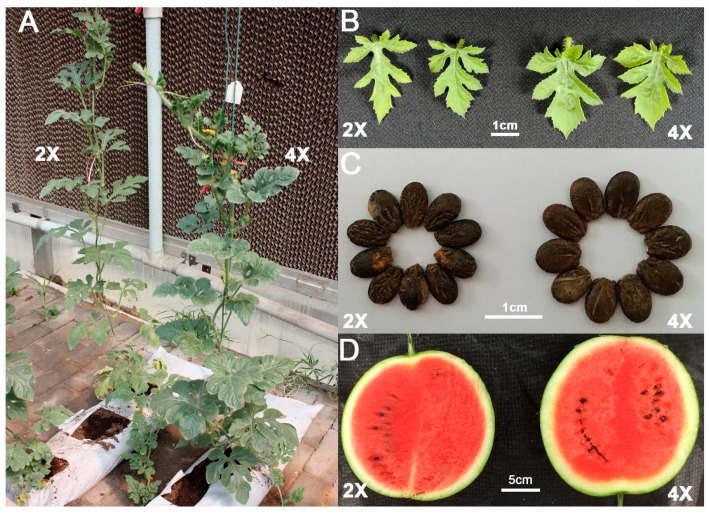
Plants (**A**), young leaves (**B**), seeds (**C**), and fruits (**D**) of diploid (E46, 2 ×) and tetraploid (yE46, 4 ×) watermelon.

**Figure 2:**
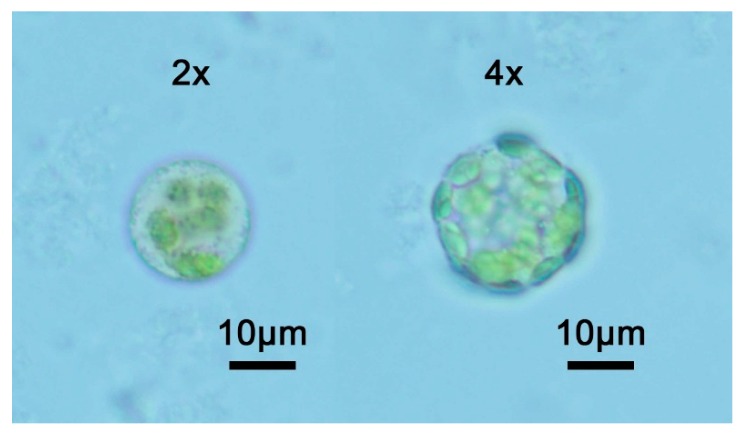
Microscopic observation of protoplasts of different ploidy watermelons. Bars 10 μm.

Diploid watermelons (E46, 2n) were induced by colchicine and a total of 20 tetraploid watermelons (yE46, 4n) were obtained. Six generations of continuous planting and self-crossing screened out high-quality watermelon. As shown in Figure 1, the tetraploid watermelon leaves, seeds, and fruits are larger than in diploid. Statistical analysis was performed on the vertical diameter, cross diameter, ratio of the fruit, and the area of the seed. Tetraploid watermelon seeds are significantly larger than diploid (Table 1). Protoplast preparation was carried out on the leaves. The results of microscopic examination and statistical analysis showed that the protoplasts of tetraploid watermelon were significantly larger than diploid (Figure 2, Table 1).

### 2.2. Tetraploid Characterization by Flow Cytometric Analysis

**Figure 3:**
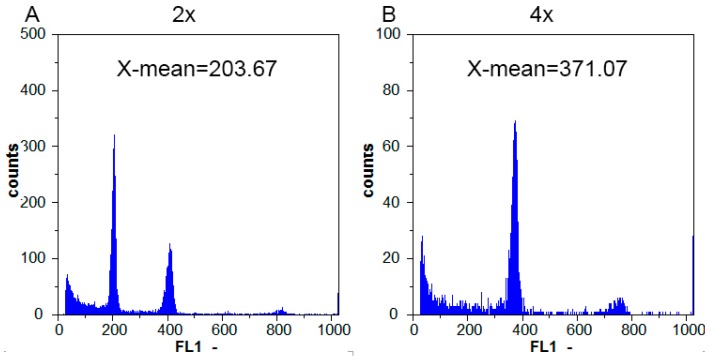
Polyploidy detection through flow cytometry. The DNA ploidy level of tetraploid watermelon (**B**, E46, 2×) was evaluated based on the DNA ploidy level of diploid watermelon (**A**, E46, 4×).

The ploidy was judged by the fluorescence intensity X-mean of the sample. The results showed that the fluorescence intensity of the main peak of diploid E46 plant was 203.46, and the fluorescence intensity of the main peak of tetraploid yE46 plant was about 371.07, which was about twice that of E46, indicating that yE46 plant cells DNA content was 1.8-times that of the E46 and was a tetraploid plant (Figure 3).

### 2.3. 5S rDNA Sequences in Watermelon Genome

**Figure 4:**
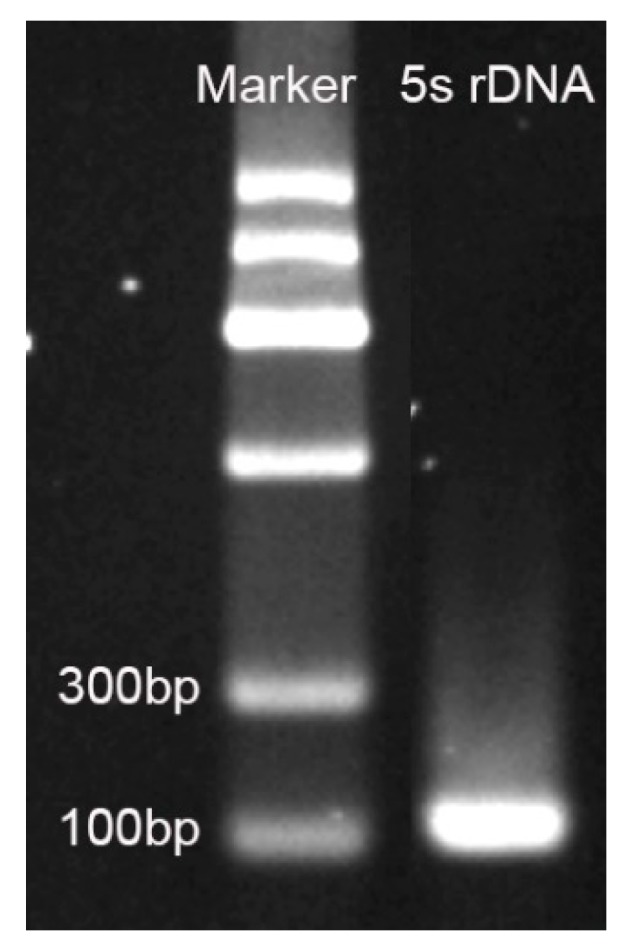
5S rDNA homologous sequence amplification results.

A fragment of about 100 bp was obtained by primer amplification (Figure 4). The fragment was TA cloned and picked for monoclonal sequencing. The result showed that the length was 111 bp and the base sequence was as follows: 5′-CGATCATACCAGCACTAATGCACCGGATCCAACCAGAACTCCGCAGTTAAGCGTGCTGGGACGAGAGTAGTACTAAGTTGGGTGACCACTTGGGAAGTCCTCGTGTTGCAC-3′

**Figure 5:**
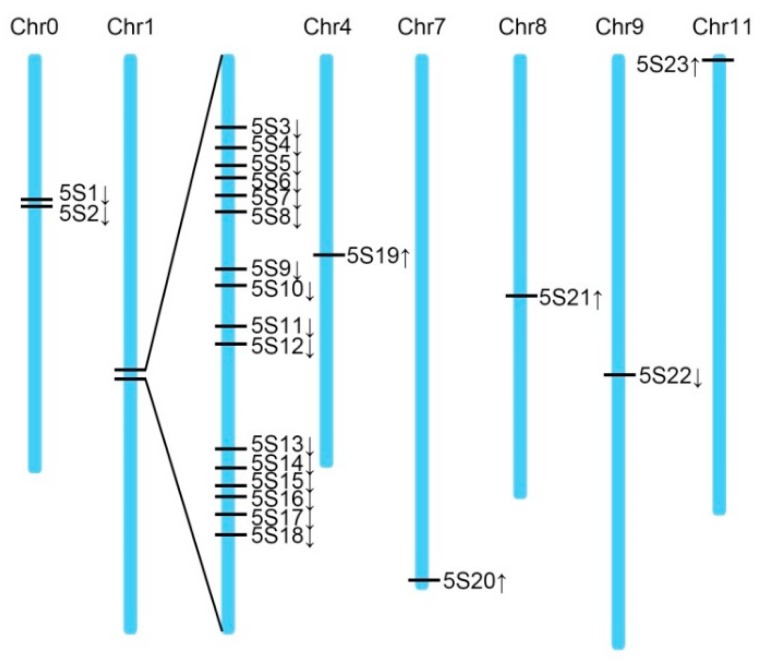
Distribution of 5S rDNA fragment homologous sequences on the chromosome.

Sequence alignment was performed using Blastn. After deleting redundant data, 23 homologous sequences of 5S rDNA fragments were obtained (Table 2), and the sequence was cloned according to its position on the chromosome. The sequence cloned in the first section was named 5S4. A physical map of the 5S rDNA fragment was drawn based on the position of each sequence on the chromosome, wherein a cluster of 16 copies was present on chromosome 1 (Figure 5).

### 2.4. Tetraploid Characterization by RT-qPCR

There are 23 copies of the 5S rDNA fragment in the watermelon genome. The watermelon genome size is known to be 425 Mb [23], and 1 ng DNA contains 9.80 × 1011 base pairs [24]. Therefore, the copy number of 5S rDNA fragments in 1 ng of watermelon DNA was 53,035.29. The above results indicate that the 5S rDNA fragment can meet the qPCR detection requirement in the lower concentration watermelon DNA solution.

**Figure 6:**
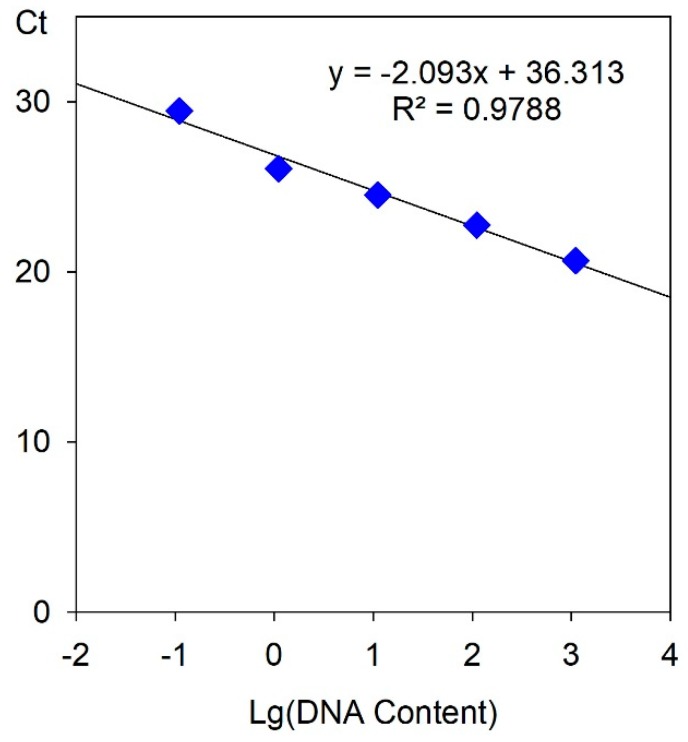
The standard curve between the Ct value in the qPCR reaction and the DNA contents in the reaction system.

The concentration of total DNA extracted was measured by NanoDrop 2000C (Thermo Scientific, Wilmington, USA), wherein the E46 DNA concentration was 110.7 ng/μL and the yE46 DNA concentration was 196.1 ng/μL, which was about 2-times different. A standard curve between the cycle threshold (Ct) value after qPCR reaction and the total amount of template in the reaction system was constructed with reference to the DNA solution of E46. 10 μL, 1 μL, 0.1 μL, 0.01 μL, and 0.001 μL of E46 DNA solution were sequentially added to different reaction systems, and the total amount of DNA in the reaction system was 1107 ng, 110.7 ng, 11.07 ng, 1.107 ng, and 0.1107 ng, respectively. After the reaction was completed, the Ct values of the respective concentration samples were converted to 20.6610, 22.7435, 24.5173, 26.0610, and 29.44673 in order to draw a standard curve, as shown in Figure 6. The regression equation is y = −2.093x + 26.875, R^2^ = 0.9788, indicating that the Ct value is highly linearly related to DNA concentration.

The results of qPCR detection of 0.2 μL of E46 and yE46 DNA solutions are shown in Table 3. After completion of the reaction, the Ct values of E46 and yE46 were 24.0873 and 23.6052, respectively, and the total amount of DNA in the E46 and yE46 reaction systems was converted according to the standard curve. They are 21.4740 ng and 36.4961 ng, respectively. The above results indicate that there is a fold relationship between the DNA concentration and the total amount in the DNA mother liquor of E46 and yE46, that is, the total amount of DNA in yE46 is about twice that of E46. The total amount of protoplasts extracted from the two sample DNAs was equal, indicating that the amount of DNA in yE46 single cells was about 1.7-times that in E46, indicating that E46 is diploid and yE46 is tetraploid. At the same time, this result is the same as the total amount of DNA measured after protoplast extraction of DNA, and consistent with the results measured by FCM.

## 3. Discussion

### 3.1. Method for Identification of Watermelon Polyploid

In the process of inducing polyploidy, accurate and timely identification of polyploid plants can shorten the culture period and improve the efficiency of polyploid breeding. Several methods such as evaluation of stomatal size, chloroplast number of the guard cells, morphological, FCM, and chromosome counts can be used to determine polyploidy, which can be used for preliminary screening and judgment of polyploid [14,25]. Polyploid plants have obvious differences in their external morphological characteristics with diploids due to chromosome doubling, mainly in the shape and size of roots, stems, leaves, flowers, and fruits. The diameter of the ovary, the petals and anther diameter of the male flower, the leaf length and the leaf width ratio are good signs of the ploidy level of the watermelon [11]. Morphological parameters are often used as the first primary selection criteria for polyploids but are generally not completely reliable [26]. For example, in *Lagerstroemia indica*, plants were selected based on morphological parameters typical for induced tetraploids (increased width-to-length leaf ratios, thicker stems, higher number of chloroplasts per guard cell, and larger stomata) and afterward confirmed by FCM [27]. Surprisingly, only 50% of the morphologically screened tetraploid plants were confirmed to be tetraploids [27]. FCM quickly detects the ploidy of a large number of plants and analyzes different types of tissues and cell layers [18,28]. It allows polyploid plants to be analyzed at an early stage, which saves space and time [29,30]. However, the FCM method requires more accurate samples, e.g., the detection is tissue-specific and cell division is under control. The technique also requires expensive equipment. Counting chromosomes is the most direct and most accurate method to identify ploidy. However, chromosome counting is time-consuming and laborious, and it is necessary to make good chromosomal compression and actively divide tissue, otherwise counting is difficult [31]. Brown compared these techniques and proposed that FCM is the most effective technique [32]. 

### 3.2. RT-qPCR Could be Used for Tetraploid Characterization

Even if the above methods can successfully detect ploidy levels [14], they all have different shortcomings. Theoretically, FCM detects DNA and correlates DNA content with ploidy numbers. Inspired with this idea, we develop a widely used qPCR method. The ribosomal DNA gene (rDNA gene) is a highly repetitive and transcriptionally active gene family in the genome of eukaryotes, and the DNA encoding 45S and 5S rRNA is the most important housekeeping gene. Its tandem organization and high copy number are specific markers for determining the chromosome and karyotype of different plant species [22,33,34]. A close relationship between rDNA copy number and genome size was verified [35]. Although the process of the qPCR method is more complicated than the FCM method, which requires three steps of enzymatic hydrolysis and DNA extraction, the qPCR method requires lower samples and is not tissue-specific. In addition, the qPCR has comparable efficiency to FCM and can perform statistical analysis on a large number of cells. In our results, these two methods are quite accurate. In addition, because it requires only conventional fluorescent quantitative PCR instruments and commercial reagents, the requirements for instruments and equipment are lower and less expensive. It is equipped in conventional laboratories and the related reagent consumables are inexpensive. In contrast, the price of the conventional flow cell instrument is much higher than that of the conventional fluorescence quantitative PCR instrument. It is widely used in medical testing. The conventional plant research laboratory is less equipped, and the price of related reagents is higher, and the daily management and maintenance is more complicated. Thus supplementing the method of watermelon ploidy identification.

## 4. Materials and Methods 

### 4.1. Tetraploid Induction and Morphological Characterization

The material is homologous diploid (E46, 2n) and tetraploid (yE46, 4n) watermelon. Tetraploid watermelon is induced by homologous diploid watermelon by colchicine (Sigma Chemical Company, MO, USA) [36], the diploid drying seeds were soaked in warm water at 50 °C for 30 min, soaked in 0.5% KMnO_4_ (Sinopharm Chemical Reagent Company, Shanghai, China) for 30 min, then washed with water and soaked in water for 6 h. Germination at 28 °C for 24–36 h. Seeded in a 72-well format tray (Grass soil:Perlite = 3:1), 25 °C. When the cotyledons of the seedlings were flattened, the new leaves of the growing point were removed, and the growth point was treated with a 0.3% colchicine, which was treated twice every day at 8:00 and 18:00 for 7 days. A total of 598 strains were treated.

The morphological characteristics of the plants in the field were observed, including leaf, seed, and fruit size. The number of chloroplasts of the lower epidermis was examined by a microscope (Olympus, Tokyo, Japan), and 10 stomatal guard cells were counted per plant to identify ploidy. The number of tetraploid strains was 35, artificial pollination followed, and finally, 20 watermelons were collected.

### 4.2. Flow Cytometric Analysis

The ploidy of E46 and yE46 was detected by FCM (Partec CyFlow Space, Muenster, Germany), and a small number of young leaves were taken and processed according to the instructions of the Partec CyStain UV Precise P kit (Partec, Muenster, Germany). The ploidy is judged by the fluorescence intensity X-mean of the sample. Taking the known diploid watermelon as a control, if the diploid peak is at 200, the tetraploid will be at 400.

### 4.3. 5S rDNA Sequence Retrieval

The 5S rDNA sequence in plants is highly conserved. Primers (Table 4) have previously reported homologous sequence cloning in the total DNA of *Agave tequila* [22]. The watermelon 5S rDNA fragment was used as a search sequence, and the watermelon genome sequence was searched to determine its copy number in the genome. Downloaded the Blast program software in the NCBI database and performed a local Blast search after installation (ftp://ftp.ncbi.nlm.nih.gov/blast/executables/blast+/LATEST/). The watermelon genome sequence was downloaded from the Cucurbitaceae genome database (http://cucurbitgenomics.org/). Sequence alignment was performed using Blastn.

### 4.4. Protoplast and DNA Isolation

Protoplasts were isolated from diploid (E46, 2 ×) and tetraploid (yE46, 4 ×) watermelon leaves (Figure 1). The leaves were grown for 2 weeks or more and were cut into 10 mm × 0.1 mm thin strips, placed in a petri dish, and 10 mL of the enzyme solution was added, and then placed in a 40 rpm shaker at 25 °C under a light-shielding condition for 4–6 h. The enzyme solution was 1.25% cellulase R10, 0.3% eductase R10, 0.4 M mannitol, 20 mM potassium chloride, and 10 mM calcium chloride, and the pH was adjusted to 5.7 [35]. After the reaction was completed, the protoplasts were collected by centrifugation at 100 g and examined by microscopy (Figure 2).

The protoplasts of different ploidy watermelons were adjusted to the same concentration by a hemocytometer, and the total DNA was extracted from an equal volume of protoplast suspension. Total DNA was extracted using the Tiangen Efficient Plant Genomic DNA Extraction Kit (DP350, Beijing, China), and the total DNA adsorbed by the adsorption column was eluted with an equal amount of TB (50 μL) buffer. The extraction process needs to strictly control the volume of the addition solution, and try to avoid the change of the total amount of DNA caused by the difference in volume of the extract.

### 4.5. RT-qPCR Analysis

The concentration of total DNA extracted from 4.4 was detected by NanoDrop 2,000C, and a standard curve between the Ct value after qPCR reaction and the total amount of template in the reaction system was constructed with reference to the DNA solution of E46. The qPCR instrument is the ABI QuantStudio 6 Flex real-time PCR system (Applied Biosystems, CA, USA), and the reaction system and procedure referred to the reported method [37]. Then, 0.2 μL of E46 and yE46 DNA solutions were used for qPCR detection to determine the Ct value of the template DNA. Each sample was set to 3 technical replicates and 3 biological replicates, and the reaction system and procedure were the same as the standard curve.

### 4.6. Statistical Analysis

Experimental data were analyzed by analysis of variance (ANOVA) and *t*-test using SPSS 11.5 (SPSS, Chicago, Illinois, USA).

## Figures and Tables

**Table 1:** Morphological comparison between diploid and tetraploid watermelon.

Genotype	Fruit			Seed Area (cm^2^)	Protoplast Area (μm^2^)
	Vertical Diameter (cm)	Cross Diameter (cm)	Aspect Ratio		
E46	16.44 ± 0.61	16.08 ± 0.44	1.02 ± 0.02	0.30 ± 0.01b	258.06 ± 10.87b
yE46	17.66 ± 0.23	17.84 ± 0.68	0.99 ± 0.03	0.37 ± 0.01a	591.02 ± 83.11a

Different letters show the significant differences between the average values according to the *t*-test at the 5% level.

**Table 2:** 5S rDNA fragment homologous sequence information.

ID	Chromosome	Location	Length (bp)
5s1	Chr0	8960268–8960378(+)	110
5s2	Chr0	8960616–8960726(+)	110
5s3	Chr1	20672252–20672358(+)	106
5s4	Chr1	20672524–20672634(+)	110
5s5	Chr1	20672797–20672907(+)	110
5s6	Chr1	20673032–20673142(+)	110
5s7	Chr1	20673317–20673424(+)	107
5s8	Chr1	20673590–20673700(+)	110
5s9	Chr1	20674311–20674421(+)	110
5s10	Chr1	20674587–20674697(+)	110
5s11	Chr1	20675133–20675243(+)	110
5s12	Chr1	20675409–20675519(+)	110
5s13	Chr1	20676875–20676985(+)	110
5s14	Chr1	20677151–20677261(+)	110
5s15	Chr1	20677427–20677537(+)	110
5s16	Chr1	20677612–20677722(+)	110
5s17	Chr1	20677885–20677995(+)	110
5s18	Chr1	20678161–20678271(+)	110
5s19	Chr4	11951587–11951479(−)	108
5s20	Chr7	30808653–30808545(−)	108
5s21	Chr8	16056311–16056201(−)	110
5s22	Chr9	23004927–23005033(+)	102
5s23	Chr11	1932259–1932150(−)	110

**Table 3:** DNA concentration measured by NanoDrop and DNA concentration converted by the qPCR method.

Sample	NanoDrop		qPCR			
	DNA Conc.	Ploidy	Ct	Total Amount of DNA	DNA Conc.	Ploidy
E46	110.7 ng/μL	2 ×	24.0873	21.4740 ng	1.0737 ng/μL	2 ×
yE46	196.1 ng/μL	4 ×	23.6052	36.4961 ng	1.8248 ng/μL	4 ×

**Table 4:** 5S rDNA homologous sequence clone primers.

Gene	Forward 5′–3′	Reverse 5′–3′	Size (bp)	Temperature (°C)	Efficiency %
5S	CGATCATACCAGCACTAAAGCACC	ATGCAACACGAGGACTTCCCAG	111	60	104
